# Clinical and morphological correlates of embryonic mosaicism: a retrospective cohort study of 10,469 blastocysts in PGT-A cycles

**DOI:** 10.3389/fendo.2026.1901424

**Published:** 2026-07-28

**Authors:** Chuanju Chen, Hao Shi, Xiao Bao, Yingpu Sun

**Affiliations:** Center for Reproductive Medicine, Henan Province Key Laboratory of Reproduction and Genetics, The First Affiliated Hospital of Zhengzhou University, Zhengzhou, China

**Keywords:** advanced maternal age, blastocyst morphology, copy number variation, mosaic embryo, preimplantation genetic testing for aneuploidy (PGT-A)

## Abstract

**Background:**

Chromosomal mosaicism presents a significant clinical and diagnostic challenge in preimplantation genetic testing for aneuploidy (PGT-A) cycles. While the meiotic origins of uniform aneuploidy are well-established, the predictors and mechanisms driving mosaicism remain less understood. This study aimed to investigate the clinical and embryological factors associated with the occurrence of mosaic embryos.

**Method:**

This retrospective cohort study analyzed 10,469 biopsied blastocysts derived from 2,484 PGT-A cycles between January 2019 and June 2024. The cohort included couples treated for advanced maternal age (AMA), recurrent miscarriage, or recurrent implantation failure. We evaluated the associations between parental baseline characteristics (e.g., age, semen quality), blastocyst morphological parameters (developmental day, expansion stage, inner cell mass [ICM] grade, and trophectoderm [TE] grade), and the incidence of mosaic embryos detected via next-generation sequencing (NGS). Multivariate GEE regression was performed to identify independent correlates.

**Results:**

The results revealed that mosaicism most frequently involved chromosomes X, 16, and 18. Upon evaluation of the biopsied cohort, multivariate analysis identified advanced maternal age as an independent factor associated with mosaic embryo formation (OR = 1.63, P < 0.01). Furthermore, suboptimal blastocyst morphological characteristics showed a strong correlation with mosaicism, including delayed development to Day 6 (OR = 1.25), lower expansion stage (stages 3 + 4 vs. 5 + 6; OR = 1.86), lower ICM grade (grade B vs. A; OR = 1.37), and lower TE grade (grade C vs. A+B; OR = 1.45) (all P < 0.05). Subgroup analyses confirmed consistent association between delayed blastulation, poor morphological scores, and a higher likelihood of mosaicism across diverse indications. Notably, unlike aneuploidy—which is predominantly associated with parental age and teratozoospermia—mosaicism primarily arises from postzygotic mitotic instability, a phenomenon uniquely correlated with lower morphological grading.

**Conclusion:**

Advanced maternal age and suboptimal blastocyst morphological development are independently and strongly associated with the occurrence of chromosomal mosaicism. These findings highlight the distinct pathophysiological mechanisms and associated clinical profiles between aneuploidy and mosaicism, providing valuable reference data for prioritizing embryo selection strategies, genetic counseling and future research in PGT-A practice.

## Introduction

With the continuous advancement of assisted reproductive technologies (ART), many infertility challenges have been alleviated. Nevertheless, implantation failure and miscarriage remain major obstacles following embryo transfer. Previous studies have demonstrated a strong association between miscarriage and chromosomal abnormalities in embryos (1, 2), particularly during early pregnancy (3). Preimplantation genetic testing for aneuploidy (PGT-A) has become a pivotal tool for improving the outcomes of embryo transfer. By applying sequencing technologies to assess chromosomal copy number, PGT-A provides a genetic basis for embryo selection and helps address recurrent implantation failure and recurrent miscarriage from a genetic perspective (4). Among various chromosomal abnormalities, aneuploid and mosaic embryos are the most common.

Mosaicism refers to the coexistence of two or more genetically distinct cell lines within the same individual. When present in embryos, this condition is defined as a mosaic embryo. Mosaicism primarily arises from mitotic errors, including chromosomal nondisjunction, anaphase lag, and chromosome replication errors (5–7) First described in 1993 (8), mosaic embryos have been reported to occur in 3%–24% of blastocysts analyzed by preimplantation genetic testing (5, 9). While the implications of aneuploid embryos for pregnancy outcomes and congenital anomalies have been extensively studied (10, 11), research on mosaic embryos remains relatively limited. Their prevalence and biological complexity have, however, attracted increasing attention from the scientific community.

Previous investigations into the factors associated with mosaic embryo formation have explored maternal age, maternal body mass index (BMI), paternal age, semen quality, fertilization method, embryo developmental stage, and the testing laboratory (9, 12, 13). However, findings to date remain inconsistent and inconclusive (9, 13–15). Notably, even with respect to the effect of maternal age, results across studies have been variable and sometimes contradictory (6, 9, 13). In summary, the mechanisms underlying mosaicism, its contributing factors, classification criteria, and clinical management strategies remain incompletely understood.

In this study, we retrospectively analyzed PGT-A cycles at our center to investigate the factors associated with the occurrence of mosaic embryos. Using multivariable regression models, we systematically evaluated the relationships between mosaicism and parameters such as maternal age, paternal age, and embryo quality. Our findings aim to provide evidence to guide clinical decision-making, with the goal of reducing the occurrence of mosaic embryos through consideration of both intrinsic parental factors and external influences, thereby contributing to a deeper understanding and refinement of ART practice.

## Materials and methods

### Study population and eligibility criteria

Clinical records of couples who underwent PGT-A at the Reproductive Medicine Center of the First Affiliated Hospital of Zhengzhou University between January 1, 2019, and June 30, 2024, were retrospectively reviewed. Clinical data were obtained from the Clinical Reproductive Medicine Management System (CCRM) of the Reproductive Medicine Center, the First Affiliated Hospital of Zhengzhou University. The study analyzed the baseline characteristics of mosaic embryos and explored factors associated with mosaicism during PGT-A cycles.

The study populations were defined according to established clinical criteria. Advanced maternal age (AMA) was determined based on the Chinese Expert Consensus on preimplantation genetic testing, which defines advanced age as ≥38 years (16). Recurrent miscarriage (RM) referred to the occurrence of three or more early pregnancy losses before 12 weeks of gestation (17). Recurrent implantation failure (RIF) was defined following both international recommendations (18) and the consensus of the Reproductive Medicine Committee of the Chinese Medical Doctor Association, as the failure to achieve a clinical pregnancy after the transfer of at least three good-quality embryos within three fresh or frozen embryo transfer cycles in women younger than 40 years. Good-quality embryos included Day 3 embryos with eight or more blastomeres, uniform blastomere size, and <10% fragmentation, as well as blastocysts graded 3BB or higher. A history of chorionic chromosomal abnormalities was assigned to couples in whom genetic testing of miscarriage tissue revealed chromosomal abnormalities in at least one previous pregnancy loss.

The eligibility criteria were as follows. Inclusion criteria: a) Patients who underwent PGT-A between January 1, 2019, and June 30, 2024, due to advanced maternal age, recurrent miscarriage, or recurrent implantation failure. b) Availability of blastocysts suitable for biopsy and diagnosis. c) Both partners had normal karyotypes. Exclusion criteria: a) Cycles involving donor sperm or donor oocytes. b) Couples with chromosomal abnormalities or monogenic disorders. c) Blastocyst biopsy performed on frozen sperm cycles or thawed embryos in frozen-thawed transfer cycles. d) Presence of severe systemic diseases, such as systemic lupus erythematosus, diabetes mellitus, or thyroid dysfunction.

### Controlled ovarian stimulation

Patients undergoing PGT-A received controlled ovarian stimulation according to the routine protocol of our center, using a short luteal-phase GnRH agonist regimen. Triptorelin was administered intramuscularly during the mid-luteal phase. After 10 days, the dose was reduced to 0.05 mg/day. Once pituitary downregulation was achieved—defined as hormone levels of FSH < 5 mIU/mL, LH < 5 mIU/mL, E2 < 30 pg/mL, P < 0.6 ng/mL, follicle diameter of 4–7 mm, and endometrial thickness < 5 mm—ovarian stimulation was initiated with follicle-stimulating hormone (FSH).

Follicular development was monitored by transvaginal ultrasound combined with serum hormone measurements. When at least one dominant follicle reached a diameter ≥ 20 mm, three follicles reached ≥ 17 mm, or two to three follicles reached ≥ 16 mm, final oocyte maturation was triggered with a combined injection of human chorionic gonadotropin (hCG) at a dose of 2000 IU and recombinant human chorionic gonadotropin at a dose of 250 μg. Oocyte retrieval was performed 36–38 hours later under intravenous anesthesia and transvaginal ultrasound guidance.

### Intracytoplasmic sperm injection

To minimize the risk of polyspermy and avoid contamination from paternal genetic material, intracytoplasmic sperm injection (ICSI) was used for fertilization in the PGT-A group. Four to six hours after oocyte retrieval, sperm were selected based on morphology and motility. Following sperm aspiration, ICSI was performed under micromanipulation. Sixteen to eighteen hours after ICSI, pronuclear formation was evaluated under an inverted microscope. The presence of two pronuclei (2PN) within the oocyte was considered indicative of normal fertilization.

### Blastocyst grading and biopsy

Blastocyst quality was assessed using the Gardner grading system (19), which evaluates embryos based on three dimensions: degree of expansion, inner cell mass, and trophectoderm morphology. Trophectoderm biopsy was performed for genetic analysis (20, 21). On day 4 of embryo culture, a small opening was made in the zona pellucida using a laser-assisted hatching system (OCTAX, Germany). Embryos were then cultured until day 5–6 for blastocyst development assessment.

For biopsy, blastocysts with partially herniated trophectoderm cells were placed in G-MOPS medium droplets within labeled biopsy dishes. Laser pulses were applied to separate trophectoderm cells, and 3–5 cells were removed. Following biopsy, blastocysts were promptly transferred into four-well plates and cultured in an incubator at 37 °C with 6% CO_2_ until cryopreservation. The numerical labeling of blastocysts in the four-well plates was required to match the corresponding biopsy dish numbers.

### Whole genome amplification and embryo aneuploidy detection

Trophectoderm (TE) cells obtained from biopsy were subjected to whole genome amplification (WGA) using the Multiple Annealing and Looping-Based Amplification Cycles (MALBAC) method (22). The amplified products were used to construct sequencing libraries, which were subsequently sequenced on either the HiSeq 2500 platform (Illumina, USA) or the BGI 200 platform (BGI, China) (diagnostic comparability between the two platforms was confirmed to be balanced). Sequencing was performed in single-end mode with a read length of 50 bp. To ensure accuracy and sufficient coverage, each sample was required to achieve a minimum of 2 Mb of sequencing reads.

Copy number variation (CNV) detection followed a standardized quality control pipeline (23). Sequencing reads were preprocessed to remove adapter sequences and low-quality bases. High-quality reads were then aligned to the human reference genome (hg19) using appropriate algorithms. After correcting for GC-content bias, normalized read counts were calculated across the genome using a sliding window of 600 Kb. To specifically mitigate the impact of background noise and WGA-induced amplification bias, a strict bioinformatic quality control (QC) threshold was implemented. Sequencing profiles exhibiting excessive basal noise—quantified by an intra-chromosomal coefficient of variation (CV) exceeding our established internal laboratory threshold—were categorized as “noisy” or “non-informative.” To prevent false-positive mosaic calls, embryos with such noisy profiles were excluded from the final analytical cohort or subjected to re-biopsy if clinically indicated. Only profiles passing these rigorous QC metrics were advanced for CNV visualization, where copy number states were plotted according to chromosome length using distinct color codes.

A copy number of 2 indicated euploidy for that chromosomal region. Copy numbers of 1 or 3 indicated aneuploidy, including monosomy, trisomy, or segmental aneuploidy. Intermediate copy numbers were interpreted as mosaicism. Using a threshold of 30%–70% as an example, embryos were classified as mosaic when copy numbers fell outside the normal diploid range (1.7–2.3) but did not meet the thresholds for monosomy or trisomy (<1.3 or >2.7). Values within the ranges of 1.3–1.7 or 2.3–2.7 were considered indicative of mosaicism (24).

### Statistical analysis

Data analyses were performed using SPSS version 25.0 (IBM, USA) and R software version 4.4.2. Continuous variables with normal distribution were expressed as mean ± standard deviation, while those with non-normal distribution were expressed as median (interquartile range). Categorical variables were summarized as proportions and percentages. Analysis of variance (F-test) was used for continuous variables, and the chi-square test (χ² test) was applied for categorical variables.

To account for the clustering structure of embryos derived from the same oocyte retrieval cycle, generalized estimating equation (GEE) models with a logit link function were applied to assess associations between clinical factors and mosaic embryo formation in PGT-A cycles. Both univariate and multivariable GEE analyses were performed. Variables with potential clinical relevance were further included in multivariable GEE models to identify independent factors associated with mosaic embryo formation. To address potential confounding effects and ensure the robustness of the multivariate models, collinearity diagnostics were performed. Variance inflation factor (VIF) was used to assess multicollinearity among covariates, with a VIF value > 5 considered indicative of problematic collinearity. Additionally, interaction testing was conducted within the GEE framework to evaluate whether the associations between morphology and mosaicism were modified by maternal age. Results were presented as odds ratios (ORs) with 95% confidence intervals (CIs). All P values were two-sided, and statistical significance was defined as *P* < 0.05. For multiple comparisons, Bonferroni correction was applied.

## Results

### Baseline characteristics of included cycles and embryo groups

A total of 2,484 PGT-A diagnostic cycles were included in this study, among which 1,652 cycles yielded euploid embryos (66.51%), while 832 cycles did not (33.49%). The baseline characteristics of the included cycles are summarized in **Supplementary Table 1**.

The baseline characteristics of the three embryo groups are presented in **Table 1**. Statistical analyses revealed significant differences among the groups in female age, anti-Müllerian hormone (AMH) level, number of oocytes retrieved, and blastocyst morphology parameters, including blastocyst expansion, inner cell mass (ICM) grade, and trophectoderm (TE) grade (all *P* < 0.05). Significant differences were also observed in male age, the presence of teratozoospermia, total gonadotropin (Gn) dose, blastocyst day, and the proportions of patients with advanced maternal age, recurrent implantation failure, and a history of chorionic chromosomal abnormalities. Pairwise comparisons further confirmed that several of these variables remained significantly different between groups (*P* < 0.05, **Table 1**).

**Table 1:** Baseline characteristics of included embryos.

Basic characteristics	Euploid group	Mosaic group	Aneuploid group	χ^2^/F	P value *
Female age (years)	32.35 ± 3.96†	33.67 ± 4.72	34.82 ± 4.89	236.32	<0.01^abc^
Female BMI (kg/m²)	23.15 ± 3.08	23.24 ± 3.05	23.36 ± 2.99	2.99	0.05
Basal FSH (mIU/mL)	6.70 ± 17.74	6.38 ± 2.15	6.46 ± 2.24	0.68	0.51
Basal LH (mIU/mL)	5.89 ± 5.41	5.77 ± 5.13	5.72 ± 5.31	0.78	0.46
Basal E_2_ (pg/mL)	276.42 ± 1055.23	328.08 ± 1226.59	266.36 ± 993.01	2.58	0.08
Basal P (ng/mL)	1.30 ± 4.01	1.24 ± 3.98	1.40 ± 4.14	0.96	0.38
AMH(ng/mL)	4.34 ± 3.74	4.13 ± 3.97	3.56 ± 2.72	30.97	<0.01^bc^
No. of oocytes retrieved	18.43 ± 8.48	17.22 ± 8.62	16.19 ± 8.06	49.93	<0.01^abc^
Blastocyst expansion stage					
Stage 3 + 4 (%)	91.19 (3747/4109)‡	94.15 (3618/3843)	94.30 (2232/2367)	34.55	<0.01^ab^
Stage 5 + 6 (%)	8.81 (362/4109) b	5.85 (225/3843)	5.70 (135/2367)		
ICM grade					
Grade A (%)	6.06 (249/4109)	3.67 (141/3845)	3.93 (93/2368)	29.39	<0.01^ab^
Grade B (%)	93.94 (3860/4109)	96.33 (3704/3845)	96.07 (2275/2368)		
TE grade					
Grade A+B (%)	58.41 (2400/4109)	46.94 (1805/3845)	43.37 (1027/2368)	170.28	<0.01^abc^
Grade C (%)	41.59 (1709/4109)	53.06 (2040/3845)	56.63 (1341/2368)		
Male age (years)	33.19 ± 4.59	34.51 ± 5.34	35.8 ± 5.79	196.29	<0.01^abc^
Teratozoospermia (%)	11.88 (307/2584)	14.20 (350/2464)	13.43 (214/1594)	6.16	0.05^a§^
Total Gn dosage (IU)	2386.56 ± 808.40	2513.13 ± 846.97	2620.96 ± 830.52	56.35	<0.01^abc^
Blastocyst day					
Day 5 (%)	58.64 (2417/4122)	52.74 (2033/3855)	49.18 (1170/2379)	60.15	<0.01^abc^
Day 6 (%)	41.36 (1705/4122)	47.26 (1822/3855)	50.82 (1209/2379)		
Subgroups					
Advanced Maternal Age	10.62 (433/4076)†	22.48 (859/3822)	31.76 (748/2355)	443.91	<0.01^abc^
Recurrent Miscarriage	21.23 (876/4127)	20.31 (783/3856)	21.25 (506/2381)	1.27	0.53
Recurrent Implantation Failure	16.67 (651/3906)	18.86 (628/3330)	16.78 (320/1907)	6.83	0.03
History of chorionic chromosomal abnormalities	40.97 (1691/4127)	39.21 (1512/3856)	42.97(1023/2381)	8.70	0.01^c^

*P values indicate overall comparisons among the three groups, For significant overall results, *post-hoc* pairwise comparisons were performed and adjusted using the Bonferroni.

^a^Significant difference between the euploid and mosaic groups.

^b^Significant difference between the euploid and aneuploid groups.

^c^Significant difference between the mosaic and aneuploid groups.

^§^P = 0.046. ^†^Continuous variables are expressed as mean ± standard deviation; ^‡^Categorical variables are presented as proportions (percentages).

### Distribution of embryonic aneuploidy detected by PGT-A

**Figure 1:**
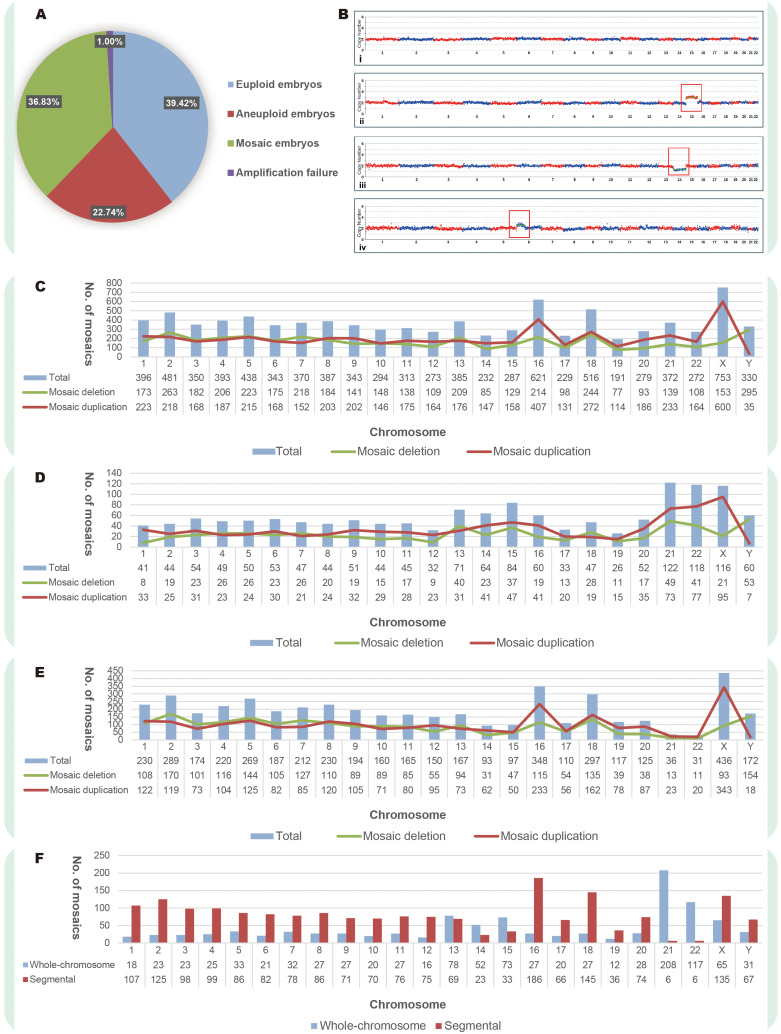
Distribution of embryo ploidy status and chromosomal characteristics of mosaicism detected by PGT-A. **(A)** A pie chart summarizing the overall ploidy status of the 10,469 embryos analyzed, categorized into euploid (39.42%), aneuploid (22.74%), mosaic (36.83%), and 41amplification failure (1.00%). **(B)** Representative copy number variation (CNV) profiles generated by next-generation sequencing (NGS). Panels (i) to (iv) illustrate distinct ploidy patterns, with red boxes highlighting the specific chromosomal regions exhibiting copy number shifts indicative of aneuploidy or mosaicism. **(C)** The overall distribution and frequency of mosaic events (total, mosaic deletions, and mosaic duplications) across individual chromosomes (1–22, X, and Y). **(D–F)** he frequency distributions of involved chromosomes in different subtypes of mosaic embryos, specifically representing whole-chromosome mosaic embryos **(D)**, segmental-chromosome mosaic embryos **(E)**, and complex mosaic embryos **(F)**. Overall, mosaic events were most frequently observed in chromosomes X, 16, and 18. Specifically, whole-chromosome mosaicism predominantly affected chromosomes 21, 22, and X, while segmental mosaicism was more prevalent in chromosomes X, 16, and 18.

A total of 10,469 embryos were analyzed in all included PGT-A cycles, among which 3,856 (36.83%) were identified as mosaic, 4,127 (39.42%) were euploid, and 2,381 (22.74%) were aneuploid. In addition, 105 embryos (1.00%) failed to be amplified (**Figure 1A**). Embryonic aneuploidy was determined based on copy number variation (CNV) analysis, and the distribution of different CNV patterns is shown in **Figure 1B**.

Among the 3,856 mosaic embryos, the frequencies of mosaicism detected in each chromosome are presented in **Figure 1C**. Based on the type of chromosomal involvement, mosaic embryos were further classified into whole-chromosome mosaic, segmental-chromosome mosaic, and complex mosaic. **Figures 1D-F** illustrate the frequency of each mosaic type across individual chromosomes. Overall, mosaic events were most frequently observed in chromosomes X, 16, and 18. Specifically, whole-chromosome mosaicism occurred more often in chromosomes 21, 22, and X, whereas segmental mosaicism was more prevalent in chromosomes X, 16, and 18.

### Analysis of factors associated with mosaic embryo formation

To explore the potential factors associated with the formation of mosaic embryos, univariate GEE regression analyses were first conducted by comparing the mosaic and aneuploid groups separately with the euploid group. The results showed that multiple variables differed significantly between groups. Compared with the euploid group, patients in the mosaic group exhibited significant differences in female age, male age, blastocyst developmental day, blastocyst expansion degree, ICM grade, TE grade, and the proportion of recurrent implantation failure (*P* < 0.05; **Supplementary Table 2**).

When comparing the aneuploid group with the euploid group, significant differences were also observed in female age, AMH, male age, total Gn dose, number of retrieved oocytes, blastocyst developmental day, expansion degree, ICM grade, and TE grade (*P* < 0.05), while other variables showed no statistically significant differences (**Supplementary Table 2**).

Subsequently, multivariate GEE regression analysis was performed, adjusting for potential confounders including advanced maternal age, paternal age, number of retrieved oocytes, blastocyst developmental day, blastocyst expansion degree, ICM grade, TE grade, total Gn dose, teratozoospermia, as well as history of recurrent miscarriage, recurrent implantation failure, and abnormal chorionic villus karyotype. The results indicated that advanced maternal age was independently associated with a higher likelihood of embryonic mosaicism (OR = 1.63, *P* < 0.01). Moreover, blastocyst expansion degree (stage 3 + 4 vs. 5 + 6; OR = 1.71, *P* < 0.01), TE grade (grade C vs. A + B; OR = 1.41, *P* < 0.01), and blastocyst developmental day (day 6 vs. day 5; OR = 1.27, *P* < 0.01) were all significantly associated with mosaic embryo formation.

In addition, multivariate analysis revealed that the occurrence of aneuploid embryos was significantly associated with increased maternal and paternal ages (*P* < 0.05). Blastocyst developmental day, blastocyst expansion degree, and TE grade also showed significant influences on the formation of aneuploid embryos (*P* < 0.05) (**Table 2**). Collinearity diagnostics confirmed that multicollinearity was negligible among the predictors in the multivariate models, as all Variance Inflation Factor (VIF) values were remarkably low (Maternal age: 1.006; Blastocyst expansion: 1.008; ICM grade: 1.031; TE grade: 1.042). Furthermore, formal interaction testing revealed no significant interactions between maternal age and morphological parameters (Age vs. Expansion, P = 0.716; Age vs. TE grade, P = 0.225), confirming that the predictive value of blastocyst morphology for mosaicism is independent of maternal age.

**Table 2:** Multivariate generalized estimating equation (GEE) analysis of factors associated with mosaic embryo formation in PGT-A cycles.

Variables	Mosaic vs. euploid		Aneuploid vs. euploid	
	OR (95% CI)	P value	OR (95% CI)	P value
Advanced maternal age	1.63 (1.24, 2.14)	<0.01	1.79 (1.33, 2.41)	<0.01
Male age	1.01 (0.99, 1.03)	0.24	1.05 (1.03, 1.07)	<0.01
Teratozoospermia	1.13 (0.91, 1.40)	0.27	1.02 (0.79, 1.31)	0.89
Total Gn dose *	0.99 (0.60, 1.65)	0.97	1.15 (0.59, 2.26)	0.68
No. of oocytes retrieved	1.00 (0.99, 1.01)	0.92	0.99 (0.98, 1.00)	0.04
Blastocyst day ^a^	1.25 (1.10, 1.44)	<0.01	1.35 (1.14, 1.59)	<0.01
Blastocyst expansion ^b^	1.86 (1.47, 2.37)	<0.01	2.02 (1.53, 2.66)	<0.01
ICM grade ^b^	1.37 (1.01, 1.86)	0.04	1.03 (0.73, 1.45)	0.87
TE grade ^b^	1.45 (1.27, 1.65)	<0.01	1.66 (1.43, 1.93)	<0.01
Recurrent miscarriage	0.95 (0.81, 1.11)	0.50	0.99 (0.81, 1.22)	0.96
Recurrent implantation failure	1.10 (0.92, 1.32)	0.29	0.94 (0.75, 1.19)	0.63
History of chorionic chromosomal abnormalities	0.91 (0.79, 1.06)	0.23	1.08 (0.90, 1.30)	0.41

OR, odds ratio; CI, confidence interval; Gn, gonadotropin; ICM, inner cell mass; TE, trophectoderm. ^a^Comparison between Day 6 and Day 5 blastocysts. ^b^Blastocyst expansion compares stages 3 + 4 vs 5 + 6; ICM grade compares grade B vs grade A; TE grade compares grade C vs grades A+B. ^*^Total Gn dose is expressed in log-transformed value.

### Subgroup analysis

In the subgroups of women with advanced maternal age, recurrent miscarriage, and recurrent implantation failure, subgroup analyses were performed. Initially, univariate GEE regression analyses were conducted to identify variables associated with the occurrence of mosaic embryos (**Supplementary Tables 3**-**5**). Variables that showed statistical significance were then included in multivariate GEE regression models to adjust for potential confounding factors.

a. Advanced maternal age

In both advanced and non-advanced maternal age subgroups, univariate GEE regression analyses were first performed to identify variables associated with mosaic embryo formation (**Supplementary Table 3**). Significant variables from the univariate models were subsequently included in multivariate GEE regression analyses to adjust for potential confounders (**Table 3**).

**Table 3:** Subgroup analysis using multivariable generalized estimating equation (GEE) of factors associated with mosaic embryo formation in advanced and non-advanced female age groups undergoing PGT-A.

Variables	Advanced age subgroup				Non-advanced age subgroup			
	Mosaic vs. euploid		Aneuploid vs. euploid		Mosaic vs. euploid		Aneuploid vs. euploid	
	OR (95% CI)	P value	OR (95% CI)	P value	OR (95% CI)	P value	OR (95% CI)	P value
Female age	1.39 (0.89, 2.15)	0.14	1.18 (0.75, 1.86)	0.48	1.02 (1.00, 1.05)	0.10	1.05 (1.01, 1.09)	<0.01
Male age	1.00 (0.94, 1.05)	0.88	1.02 (0.97, 1.08)	0.45	1.00 (0.98, 1.02)	0.96	1.02 (1.00, 1.04)	0.10
Total Gn dose *	0.62 (0.07, 5.24)	0.66	3.42 (0.34, 34.03)	0.29	0.88 (0.56, 1.38)	0.58	0.98 (0.53, 1.80)	0.94
No. of oocytes retrieved	0.97 (0.94, 1.01)	0.10	1.00 (0.97, 1.03)	0.99	1.00 (0.99, 1.01)	0.84	0.99 (0.98, 1.00)	0.13
Blastocyst day ^a^	1.11 (0.76, 1.61)	0.59	1.25 (0.81, 1.92)	0.31	1.20 (1.06, 1.35)	<0.01	1.32 (1.14, 1.53)	<0.01
Blastocyst expansion ^b^	1.66 (0.84, 3.28)	0.14	3.52 (1.53, 8.10)	<0.01	1.82 (1.47, 2.25)	<0.01	1.89 (1.48, 2.43)	<0.01
ICM grade ^b^	0.53 (0.21, 1.33)	0.18	0.69 (0.27, 1.73)	0.42	1.50 (1.15, 1.95)	<0.01	1.24 (0.90, 1.71)	0.19
TE grade ^b^	1.66 (1.18, 2.34)	<0.01	1.46 (1.01, 2.11)	0.04	1.47 (1.31, 1.64)	<0.01	1.66 (1.45, 1.90)	<0.01
Recurrent miscarriage	0.76 (0.46, 1.26)	0.30	0.70 (0.41, 1.18)	0.18	1.00 (0.87, 1.15)	0.99	0.98 (0.81, 1.19)	0.86
Recurrent implantation failure	1.20 (0.64, 2.23)	0.57	1.03 (0.53, 1.98)	0.94	1.11 (0.94, 1.30)	0.22	1.05 (0.85, 1.29)	0.66
History of chorionic chromosomal abnormalities	1.43 (0.92, 2.23)	0.11	1.03 (0.64, 1.66)	0.89	0.88 (0.77, 1.00)	0.05	1.15 (0.97, 1.36)	0.10

OR, odds ratio; CI, confidence interval. ^a^Comparison between Day 6 and Day 5 blastocysts. ^b^Blastocyst expansion compares stages 3 + 4 vs 5 + 6; ICM grade compares grade B vs grade A; TE grade compares grade C vs grades A+B. ^*^Total Gn dose is expressed in log-transformed values.

In the advanced maternal age subgroup, a lower TE grade (grade C vs. grades A+B, OR = 1.66, *P* < 0.01) remained significantly associated with the occurrence of mosaic embryos. In the non-advanced maternal age subgroup, day-6 blastocysts (vs. day 5, OR = 1.20, *P* < 0.01), lower blastocyst expansion stage (stages 3 + 4 vs. 5 + 6, OR = 1.82, *P* < 0.01), lower ICM grade (grade B vs. grade A, OR = 1.50, *P* < 0.01), and lower TE grade (grade C vs. grades A+B, OR = 1.47, *P* < 0.01) were all significantly associated with mosaic embryo formation, whereas other variables showed no significant differences after adjustment.

b. Recurrent miscarriage

Among women with and without recurrent miscarriage, univariate GEE regression analyses were first performed (**Supplementary Table 4**), and variables with statistical significance were subsequently entered into multivariate GEE models (**Table 4**).

**Table 4:** Subgroup analysis using multivariable generalized estimating equation (GEE) of factors associated with mosaic embryo formation in patients with and without recurrent miscarriage undergoing PGT-A.

Variables	Recurrent miscarriage subgroup				Non-recurrent miscarriage subgroup			
	Mosaic vs. euploid		Aneuploid vs. euploid		Mosaic vs. euploid		Aneuploid vs. euploid	
	OR (95% CI)	P value	OR (95% CI)	P value	OR (95% CI)	P value	OR (95% CI)	P value
Advanced maternal age	1.06 (0.99, 1.14)	0.09	1.05 (0.96, 1.14)	0.31	1.74 (1.29, 2.35)	<0.01	1.96 (1.40, 2.75)	<0.01
Male age	1.02 (0.96, 1.08)	0.49	1.05 (0.98, 1.13)	0.18	1.00 (0.98, 1.02)	0.88	1.04 (1.02, 1.07)	<0.01
Teratozoospermia	1.05 (0.65, 1.70)	0.85	0.92 (0.54, 1.56)	0.75	1.16 (0.91, 1.48)	0.24	1.04 (0.78, 1.40)	0.78
No. of oocytes retrieved	0.99 (0.97, 1.01)	0.36	0.98 (0.96, 1.00)	0.12	1.00 (0.99, 1.01)	0.81	0.99 (0.97, 1.00)	0.10
Total Gn dose *	0.25 (0.09, 0.70)	<0.01	0.87 (0.20, 3.83)	0.85	1.36 (0.76, 2.45)	0.30	1.21 (0.56, 2.61)	0.62
Blastocyst day ^a^	1.71 (1.27, 2.31)	<0.01	1.38 (0.96, 1.99)	0.08	1.14 (0.98, 1.33)	0.08	1.35 (1.12, 1.63)	<0.01
Blastocyst expansion ^b^	2.03 (1.24, 3.33)	<0.01	2.34 (1.24, 4.41)	<0.01	1.82 (1.39, 2.40)	<0.01	1.95 (1.43, 2.65)	<0.01
ICM grade ^b^	1.94 (0.97, 3.86)	0.06	0.63 (0.34, 1.17)	0.14	1.28 (0.91, 1.79)	0.15	1.29 (0.85, 1.95)	0.24
TE grade ^b^	1.09 (0.84, 1.43)	0.52	1.14 (0.85, 1.52)	0.39	1.59 (1.37, 1.84)	<0.01	1.88 (1.58, 2.24)	<0.01
Recurrent implantation failure	1.28 (0.79, 2.08)	0.32	0.94 (0.38, 2.35)	0.90	1.09 (0.90, 1.32)	0.38	0.92 (0.72, 1.18)	0.50
History of chorionic chromosomal abnormalities	0.97 (0.75, 1.27)	0.83	1.31 (0.91, 1.89)	0.15	0.90 (0.76, 1.07)	0.24	1.01 (0.81, 1.26)	0.93

OR, odds ratio; CI, confidence interval. ^a^Comparison between Day 6 and Day 5 blastocysts. ^b^Blastocyst expansion compares stages 3 + 4 vs 5 + 6; ICM grade compares grade B vs grade A; TE grade compares grade C vs grades A+B. ^*^Total Gn dose is expressed in log-transformed values.

In the recurrent miscarriage subgroup, day-6 blastocysts (vs. day 5, OR = 1.71, *P* < 0.01), lower blastocyst expansion stage (stages 3 + 4 vs. 5 + 6, OR = 2.03, *P* < 0.01), and a lower total Gn dose (log-transformed) (OR = 0.25, *P* < 0.01) were significantly associated with mosaic embryo formation. In the non-recurrent miscarriage subgroup, advanced maternal age (OR = 1.74, *P* < 0.01), lower blastocyst expansion stage (OR = 1.82, *P* < 0.01), and lower TE grade (OR = 1.59, *P* < 0.01) remained significantly associated with mosaic embryo formation. No other variables demonstrated significant associations after adjustment.

c. Recurrent implantation failure

In the subgroups with and without recurrent implantation failure, univariate GEE analyses were first conducted to identify potentially relevant factors, followed by multivariate analyses adjusting for confounding variables (**Supplementary Table 5**; **Table 5**).

**Table 5:** Subgroup analysis using multivariable generalized estimating equation (GEE) of factors associated with mosaic embryo formation in patients with and without recurrent implantation failure undergoing PGT-A.

Variables	Recurrent implantation failure subgroup†				Non-recurrent implantation failure subgroup			
	Mosaic vs. euploid		Aneuploid vs. euploid		Mosaic vs. euploid		Aneuploid vs. euploid	
	OR (95% CI)	P value	OR (95% CI)	P value	OR (95% CI)	P value	OR (95% CI)	P value
Advanced maternal age	2.18 (1.13, 4.19)	0.02	3.17 (1.49, 6.78)	<0.01	1.63 (1.21, 2.19)	<0.01	1.70 (1.23, 2.35)	<0.01
Male age	1.00 (0.97, 1.03)	0.99	0.98 (0.94, 1.03)	0.51	1.01 (0.99, 1.03)	0.27	1.06 (1.03, 1.09)	<0.01
Teratozoospermia	1.05 (0.60, 1.84)	0.86	1.52 (0.80, 2.90)	0.20	1.13 (0.90, 1.43)	0.28	0.92 (0.70, 1.22)	0.56
Total Gn dose *	2.77 (1.03, 7.48)	0.04	2.21 (0.50, 9.81)	0.30	0.73 (0.41, 1.29)	0.28	1.07 (0.50, 2.29)	0.87
No. of oocytes retrieved	1.00 (0.99, 1.02)	0.65	0.97 (0.94, 1.00)	0.03	1.00 (0.99, 1.01)	0.54	0.99 (0.98, 1.00)	0.20
Blastocyst day ^a^	1.22 (0.88, 1.70)	0.24	1.07 (0.71, 1.60)	0.74	1.27 (1.09, 1.47)	<0.01	1.40 (1.16, 1.68)	<0.01
Blastocyst expansion ^b^	3.77 (2.00, 7.12)	<0.01	2.70 (1.22, 6.00)	0.01	1.71 (1.32, 2.22)	<0.01	1.92 (1.43, 2.58)	<0.01
ICM grade ^b^	2.08 (0.96, 4.51)	0.06	1.68 (0.62, 4.56)	0.31	1.27 (0.90, 1.78)	0.17	0.96 (0.66, 1.37)	0.81
TE grade ^b^	1.75 (1.27, 2.42)	<0.01	1.92 (1.25, 2.95)	<0.01	1.41 (1.22, 1.62)	<0.01	1.62 (1.38, 1.90)	<0.01
Recurrent miscarriage	2.09 (1.00, 4.35)	0.05	1.18 (0.39, 3.52)	0.77	0.94 (0.80, 1.11)	0.44	1.00 (0.80, 1.23)	0.97
History of chorionic chromosomal abnormalities	1.00 (0.57, 1.75)	0.99	0.97 (0.36, 2.61)	0.96	0.91 (0.78, 1.05)	0.20	1.09 (0.90, 1.32)	0.37

OR, odds ratio; CI, confidence interval. †Subgrouping was based on univariate GEE regression; E_2_ levels were adjusted in the multivariable model. ^a^Comparison between Day 6 and Day 5 blastocysts. ^b^Blastocyst expansion compares stages 3 + 4 vs 5 + 6; ICM grade compares grade B vs grade A; TE grade compares grade C vs grades A+B. ^*^Total Gn dose is expressed in log-transformed values.

In the recurrent implantation failure subgroup, advanced maternal age (OR = 2.18, *P* = 0.02), lower basal E_2_ level (OR = 0.65, *P* < 0.01), lower blastocyst expansion stage (stages 3 + 4 vs. 5 + 6, OR = 3.77, *P* < 0.01), and lower TE grade (grade C vs. grades A+B, OR = 1.75, *P* < 0.01) were significantly associated with mosaic embryo formation. In the non-recurrent implantation failure subgroup, advanced maternal age (OR = 1.63, *P* < 0.01), day-6 blastocysts (vs. day 5, OR = 1.27, *P* < 0.01), lower blastocyst expansion stage (OR = 1.71, *P* < 0.01), and lower TE grade (OR = 1.41, *P* < 0.01) also showed significant associations with mosaic embryo formation. Other variables did not remain significant after adjustment.

## Discussion

The development and evolution of genetic testing technologies in PGT-A have greatly expanded the understanding of embryonic chromosomal status. Mosaicism represents a unique and intriguing biological phenomenon in reproductive medicine and genetics. While commonly observed in miscarriage tissues, mosaicism has become increasingly recognized in blastocyst biopsy specimens with the advancement of preimplantation genetic testing technologies. Mosaic embryos are closely associated with ART outcomes and fetal prognosis (25, 26), underscoring their clinical importance and motivating the present investigation. In the present cohort, the overall incidence of embryonic mosaicism reached 36.83%, which is higher than the rates reported in some previous studies (25, 27–29). This variance is inextricably linked to the enhanced sensitivity of modern NGS platforms (30). Furthermore, prior research has demonstrated that the prevalence of mosaicism is highly center-specific, being significantly influenced by laboratory-specific physicochemical conditions and variations in biopsy techniques (15, 31). Our data demonstrates that different types of mosaicism tend to involve different chromosomes. Abnormal sister chromatid segregation is thought to primarily contribute to whole-chromosome mosaicism (5), whereas segmental mosaicism is associated with DNA double-strand breaks, which may occur at non-random hotspots (32). The specific mechanisms underlying the higher frequency of mosaicism in certain chromosomes, however, remain to be fully elucidated.

In the analysis of the overall population, maternal age emerged as the most significant independent factor associated with mosaic embryo formation. We observed that the odds of mosaicism were approximately 1.63-fold higher among women of advanced age. This association is biologically plausible, given that advanced maternal age is linked to compromised spindle integrity in oocytes (33, 34), reduced DNA repair capacity and diminished self-correction mechanisms (35), and dysregulation of early cell cycle processes. Unlike aneuploidy, which largely reflects meiotic errors, mosaicism primarily arises from mitotic chromosome segregation errors occurring after fertilization (36). This mechanistic distinction may explain why advanced maternal age is associated with a higher likelihood of not only aneuploidy but also mosaicism.

Beyond maternal age, another important finding of our study is the strong association between blastocyst morphologic characteristics—represented by the three components of the Gardner grading system—and the likelihood of mosaicism. Lower degrees of blastocyst expansion, reduced ICM grade, and poorer TE grade all independently increased the odds of mosaic embryo formation. These observations indicate that blastocyst morphology reflects not only developmental potential but may also mirror underlying genomic instability and uneven proliferation among cell populations. Previous studies similarly reported that higher-quality TE and more advanced expansion stages are associated with a reduced rate of *de novo* chromosomal abnormalities (37), and that Day 6 blastocysts exhibit higher rates of mosaicism than Day 5 blastocysts (38). Delayed blastulation may indicate spindle abnormalities, mitochondrial dysfunction, or gene expression dysregulation (39–41). Collectively, these results suggest that mosaicism is more likely to occur in slower-developing embryos with lower morphological scores, and that its formation may involve early cell-cycle dysregulation and imbalances in lineage allocation.

Our comparison of mosaic and aneuploid embryos further revealed distinct patterns of associated factors. Although both abnormalities were linked to embryo quality, their correlative profiles differed. Aneuploidy was more strongly associated with parental age and male teratozoospermia, whereas the correlation with ICM grade and blastocyst day were more pronounced for mosaicism. Biologically, advanced paternal age affects pre-zygotic gamete integrity leading to meiotic aneuploidy, but exerts minimal impact on the post-zygotic mitotic errors causing mosaicism. These distinctions support the notion that aneuploidy primarily originates from meiotic nondisjunction, whereas mosaicism reflects postzygotic mitotic instability. Differentiating these mechanisms provides important insights into the biological basis of chromosomal abnormalities.

The subgroup analyses among common PGT-A indications—including advanced maternal age, recurrent miscarriage, and recurrent implantation failure—showed that most factors associated with mosaicism remained consistent across subgroups. This suggests that maternal characteristics and blastocyst morphology demonstrate a robust and clinically meaningful correlation with mosaicism. To our knowledge, this is the first study to systematically compare mosaicism across three routinely targeted PGT-A subpopulations, highlighting that these correlative profiles may vary with patient background and providing valuable evidence for developing more individualized PGT-A strategies.

A major strength of our study lies in its large sample size and the inclusion of multiple clinically relevant subgroups, allowing for a comprehensive evaluation of population heterogeneity and interactions among these associated variables. We also accounted for a wide range of maternal and paternal clinical characteristics, thereby minimizing potential confounding. Furthermore, all procedures were conducted within a single center using a uniform biopsy protocol and NGS platform, ensuring high consistency and accuracy in genetic testing results.

Nonetheless, several limitations should be acknowledged. First, the generalizability of our findings is inherently limited by the study’s single-center design and its highly specific patient cohort. Our analysis exclusively included infertile couples undergoing PGT-A for strict clinical indications—specifically advanced maternal age (AMA), recurrent miscarriage (RM), and recurrent implantation failure (RIF). Therefore, it must be strongly emphasized that the incidence of mosaicism and the associated factors identified in this high-risk demographic may not directly extrapolate to standard IVF populations lacking these specific indications, nor to the general fertile population. Second, all genetic diagnoses were based on TE biopsy, which samples only 3–5 TE cells. Although concordance between TE and ICM has been reported to reach 98–100% (25, 42), discrepancies between the two cell lineages remain possible (43). Furthermore, the inherent technical limitations of TE biopsy and NGS-based mosaicism detection must not be underestimated. Because the diagnosis relies on a minimal cell number, it is highly sensitive to technical variations that can result in false-positive mosaic calls. It is imperative to distinguish true “biological mosaicism” (e.g., confined placental mosaicism) from “technical mosaicism.” The latter can frequently arise from technical confounders such as sampling bias, reciprocal errors, whole-genome amplification (WGA)-induced artifacts, S-phase artifacts (44, 45), or algorithmic smoothing masks. Consequently, the reported incidence of mosaicism in our study may encompass an interplay of both biological realities and technical artifacts.

Mosaicism likely results from a complex interplay of factors (46). The present study primarily focuses on the factors associated with mosaic embryo formation rather than analyzing the outcomes of mosaic embryo transfers. Consequently, our findings identify clinical and morphological correlates of mosaicism, rather than direct predictors of reproductive success. Nevertheless, understanding the factors associated with mosaic embryo formation remains crucial for optimizing clinical management and improving embryo selection strategies, particularly when considering the transfer of mosaic embryos. A deeper understanding of the mechanisms leading to mosaicism can help refine indications for PGT-A testing and provide a stronger evidence base for subsequent embryo-transfer decisions and clinical counseling. Future multi-center, prospective studies are needed to further elucidate the mechanisms underlying mosaic embryo formation and to clarify its implications for clinical outcomes.

## Data Availability

The raw data supporting the conclusions of this article will be made available by the authors, without undue reservation.

## Supplementary material

Supplementary Table 1 Baseline characteristics of the included PGT-A cycles.

Supplementary Table 2 Univariate generalized estimating equation (GEE) regression analysis of factors associated with mosaic embryo formation in PGT-A cycles.

Supplementary Table 3 Univariate generalized estimating equation (GEE) regression analysis of factors associated with mosaic embryo formation in advanced and non-advanced female age groups undergoing PGT-A.

Supplementary Table 4 Univariate generalized estimating equation (GEE) regression analysis of factors associated with mosaic embryo formation in patients with and without recurrent miscarriage undergoing PGT-A.

Supplementary Table 5 Univariate generalized estimating equation (GEE) regression analysis of factors associated with mosaic embryo formation in patients with and without recurrent implantation failure undergoing PGT-A.
