# Supplementary material for: Clinical and morphological correlates of embryonic mosaicism: a retrospective cohort study of 10,469 blastocysts in PGT-A cycles
Source: Front Endocrinol (Lausanne). 2026 Jul 28;17:1901424. doi: 10.3389/fendo.2026.1901424 (PMC13457482; doi:10.3389/fendo.2026.1901424)
Supplement: Supplementary file 1 [file SupplementaryFile1.docx]

**Supplementary materials**

Supplementary Table 1 Baseline characteristics of the included PGT-A cycles

| Basic Characteristics | PGT-A Cycles |
| --- | --- |
| Total PGT-A cycles (n) | 2484 |
| Cycles with euploid embryos (%) | 66.51 (1652/2484) |
| Female age (years) | 34.82±4.836 |
| Female BMI (kg/m²) | 23.16±3.002 |
| No. of oocytes retrieved | 13.50±7.639 |
| Total Gn dosage (IU) | 2764.52±845.819 |
| Female basal endocrine profile |  |
| Basal FSH（mIU/mL） | 7.10±16.375^†^ |
| Basal LH（mIU/mL） | 5.37±4.935 |
| Basal E_2_（pg/mL） | 253.02±922.285 |
| Basal P（ng/mL） | 1.28±3.924 |
| AMH（ng/mL） | 3.12±2.76 |
| Male age (years) | 35.67±5.708 |
| Teratozoospermia (%) | 14.84 (235/1584)^‡^ |
| Advanced Maternal Age（%） | 30.64 (761/2484) |
| Recurrent Miscarriage（%） | 19.85 (493/2484) |
| Recurrent Implantation Failure（%） | 18.72 (370/1976) |
| History of chorionic  chromosomal abnormalities（%） | 40.90 (1016/2484) |

Note: † Continuous variables are expressed as mean ± standard deviation; ‡ Categorical variables are expressed as percentages (proportions).

Supplementary Table 2 Univariate Generalized Estimating Equation (GEE) Regression Analysis of Factors Associated with Mosaic Embryo Formation in PGT-A Cycles

| Variables | Mosaic vs. Euploid | | Aneuploid vs. Euploid | |
| --- | --- | --- | --- | --- |
|  | OR (95% CI) | *P* value | OR (95% CI) | *P* value |
| Advanced maternal age | 1.28 (1.14, 1.43) | <0.01 | 2.37 (2.08, 2.69) | <0.01 |
| Female BMI | 1.00 (0.98, 1.01) | 0.89 | 1.02 (1.00, 1.04) | 0.07 |
| Basal FSH | 1.00 (0.99, 1.00) | 0.10 | 1.00 (1.00, 1.00) | 0.54 |
| Basal LH | 1.00 (0.99, 1.01) | 0.94 | 1.00 (0.98, 1.01) | 0.62 |
| Basal E_2_^*^ | 1.05 (0.96, 1.15) | 0.29 | 0.97 (0.86, 1.10) | 0.68 |
| Basal P | 0.99 (0.98, 1.01) | 0.24 | 1.01 (0.99, 1.02) | 0.29 |
| AMH | 1.00 (0.99, 1.02) | 0.62 | 0.92 (0.90, 0.95) | <0.01 |
| Male age | 1.01 (1.00, 1.02) | <0.01 | 1.07 (1.06, 1.08) | <0.01 |
| Teratozoospermia | 1.17 (0.97, 1.40) | 0.10 | 1.03 (0.83, 1.27) | 0.81 |
| Total Gn dosage ^*^ | 1.38 (0.98, 1.94) | 0.06 | 4.28 (2.75, 6.66) | <0.01 |
| No. of oocytes retrieved | 0.99 (0.99, 1.00) | 0.09 | 0.97 (0.97, 0.98) | <0.01 |
| Blastocyst day ^a^ | 1.09 (1.01, 1.19) | 0.04 | 1.29 (1.18, 1.41) | <0.01 |
| Blastocyst expansion stage ^b^ | 1.35 (1.15, 1.59) | <0.01 | 1.28 (1.07, 1.52) | <0.01 |
| ICM grade ^b^ | 1.38 (1.14, 1.68) | <0.01 | 1.24 (1.00, 1.54) | 0.05 |
| TE grade ^b^ | 1.29 (1.19, 1.40) | <0.01 | 1.41 (1.29, 1.54) | <0.01 |
| Recurrent Miscarriage | 0.92 (0.82, 1.04) | 0.19 | 1.03 (0.89, 1.19) | 0.71 |
| Recurrent Implantation Failure | 1.16 (1.02, 1.31) | 0.02 | 0.97 (0.83, 1.14) | 0.73 |
| History of chorionic  chromosomal abnormalities | 0.91 (0.83, 1.01) | 0.07 | 1.10 (0.98, 1.24) | 0.11 |

Notes: OR, odds ratio; CI, confidence interval; Gn, gonadotropin; ICM, inner cell mass; TE, trophectoderm.

ᵃ Comparison between Day 6 and Day 5 blastocysts. ᵇBlastocyst expansion compares stages 3+4 vs 5+6; ICM grade compares grade B vs grade A; TE grade compares grade C vs grades A+B. ^*^ E_2_ level and Total Gn dose is expressed in log-transformed values.

Supplementary Table 3 Univariate Generalized Estimating Equation (GEE) Regression Analysis of Factors Associated with Mosaic Embryo Formation in Advanced and Non-Advanced Female Age Groups Undergoing PGT-A

| Variables | Advanced Age Subgroup | | | | Non-Advanced Age Subgroup | | | |
| --- | --- | --- | --- | --- | --- | --- | --- | --- |
|  | Mosaic vs. Euploid | | Aneuploid vs. Euploid | | Mosaic vs. Euploid | | Aneuploid vs. Euploid | |
|  | OR (95% CI) | *P* value | OR (95% CI) | *P* value | OR (95% CI) | *P* value | OR (95% CI) | *P* value |
| Female age | 1.06 (1.01, 1.12) | 0.03 | 1.10 (1.05, 1.16) | <0.01 | 1.00 (0.98, 1.02) | 0.90 | 1.06 (1.03, 1.09) | <0.01 |
| Female BMI | 1.01 (0.98, 1.05) | 0.46 | 0.99 (0.95, 1.03) | 0.52 | 0.99 (0.97, 1.01) | 0.19 | 1.01 (0.99, 1.03) | 0.46 |
| Basal FSH | 0.97 (0.94, 1.01) | 0.12 | 1.04 (1.00, 1.08) | 0.04 | 1.00 (0.99, 1.00) | 0.09 | 0.98 (0.94, 1.01) | 0.23 |
| Basal LH | 1.00 (0.98, 1.02) | 0.68 | 0.99 (0.96, 1.01) | 0.35 | 1.00 (0.99, 1.01) | 0.97 | 1.00 (0.99, 1.02) | 0.58 |
| Basal E_2_^*^ | 1.09 (0.90, 1.32) | 0.38 | 1.03 (0.83, 1.28) | 0.81 | 1.04 (0.94, 1.15) | 0.44 | 0.94 (0.81, 1.09) | 0.44 |
| Basal P | 0.97 (0.94, 1.00) | 0.02 | 1.02 (0.98, 1.05) | 0.35 | 1.00 (0.98, 1.01) | 0.66 | 1.01 (0.99, 1.02) | 0.46 |
| AMH | 0.97 (0.91, 1.04) | 0.42 | 0.99 (0.93, 1.05) | 0.66 | 1.02 (1.00, 1.03) | 0.02 | 0.95 (0.93, 0.98) | <0.01 |
| Male age | 1.01 (0.99, 1.03) | 0.37 | 1.04 (1.02, 1.07) | <0.01 | 1.00 (0.99, 1.01) | 0.84 | 1.04 (1.03, 1.06) | <0.01 |
| Teratozoospermia | 1.28 (0.92, 1.77) | 0.14 | 0.81 (0.57, 1.15) | 0.24 | 1.09 (0.87, 1.37) | 0.43 | 1.09 (0.83, 1.42) | 0.54 |
| Total Gn dosage ^*^ | 1.49 (0.51, 4.37) | 0.46 | 1.28 (0.41, 3.99) | 0.68 | 0.96 (0.65, 1.42) | 0.85 | 1.95 (1.16, 3.28) | 0.01 |
| No. of oocytes retrieved | 0.98 (0.97, 1.00) | 0.04 | 1.00 (0.98, 1.02) | 0.79 | 1.00 (0.99, 1.01) | 0.61 | 0.99 (0.98, 0.99) | <0.01 |
| Blastocyst day ^a^ | 0.99 (0.82, 1.20) | 0.91 | 1.20 (0.99, 1.45) | 0.06 | 1.11 (1.01, 1.22) | 0.03 | 1.29 (1.16, 1.44) | <0.01 |
| Blastocyst expansion stage ^b^ | 1.17 (0.80, 1.70) | 0.42 | 1.53 (1.02, 2.29) | 0.04 | 1.38 (1.15, 1.65) | <0.01 | 1.16 (0.94, 1.43) | 0.16 |
| ICM grade ^b^ | 1.14 (0.72, 1.81) | 0.56 | 1.15 (0.73, 1.81) | 0.54 | 1.43 (1.16, 1.78) | <0.01 | 1.28 (0.98, 1.66) | 0.07 |
| TE grade ^b^ | 1.23 (1.03, 1.48) | 0.03 | 1.21 (1.02, 1.45) | 0.03 | 1.29 (1.17, 1.41) | <0.01 | 1.44 (1.30, 1.60) | <0.01 |
| Recurrent miscarriage | 0.88 (0.68, 1.14) | 0.34 | 0.97 (0.74, 1.28) | 0.84 | 0.94 (0.82, 1.07) | 0.33 | 1.05 (0.88, 1.26) | 0.56 |
| Recurrent implantation failure | 1.06 (0.70, 1.62) | 0.77 | 1.03 (0.64, 1.66) | 0.91 | 1.17 (1.02, 1.33) | 0.02 | 0.99 (0.83, 1.16) | 0.86 |
| History of chorionic  chromosomal abnormalities | 1.20 (0.97, 1.47) | 0.09 | 0.93 (0.74, 1.15) | 0.50 | 0.85 (0.76, 0.95) | <0.01 | 1.21 (1.05, 1.40) | <0.01 |

Notes: OR, odds ratio; CI, confidence interval; Gn, gonadotropin; ICM, inner cell mass; TE, trophectoderm.

ᵃ Comparison between Day 6 and Day 5 blastocysts. ᵇBlastocyst expansion compares stages 3+4 vs 5+6; ICM grade compares grade B vs grade A; TE grade compares grade C vs grades A+B. * E2 level and Total Gn dose is expressed in log-transformed values.

Supplementary Table 4 Univariate Generalized Estimating Equation (GEE) Regression Analysis of Factors Associated with Mosaic Embryo Formation in Patients With and Without Recurrent Miscarriage Undergoing PGT-A

| Variables | Recurrent Miscarriage Subgroup | | | | Non-Recurrent Miscarriage Subgroup | | | |
| --- | --- | --- | --- | --- | --- | --- | --- | --- |
|  | Mosaic vs. Euploid | | Aneuploid vs. Euploid | | Mosaic vs. Euploid | | Aneuploid vs. Euploid | |
|  | OR (95% CI) | *P* value | OR (95% CI) | *P* value | OR (95% CI) | *P* value | OR (95% CI) | *P* value |
| Female age | 1.03 (1.00, 1.05) | 0.05 | 1.09 (1.05, 1.13) | <0.01 | 1.02 (1.01, 1.03) | <0.01 | 1.10 (1.08, 1.11) | <0.01 |
| Female BMI | 0.98 (0.95, 1.02) | 0.39 | 1.02 (0.97, 1.06) | 0.47 | 1.00 (0.99, 1.02) | 0.75 | 1.02 (1.00, 1.04) | 0.09 |
| Basal FSH | 1.00 (0.99, 1.00) | 0.02 | 1.00 (0.99, 1.00) | 0.08 | 1.00 (0.98, 1.03) | 0.76 | 1.03 (1.01, 1.06) | 0.02 |
| Basal LH | 1.02 (0.99, 1.05) | 0.31 | 0.97 (0.94, 1.00) | 0.07 | 1.00 (0.99, 1.01) | 0.48 | 1.00 (0.99, 1.01) | 0.99 |
| Basal E_2_^*^ | 1.15 (0.95, 1.41) | 0.16 | 0.87 (0.64, 1.16) | 0.34 | 1.02 (0.93, 1.13) | 0.64 | 1.00 (0.87, 1.14) | 0.97 |
| Basal P | 1.00 (0.96, 1.04) | 0.96 | 1.01 (0.97, 1.05) | 0.65 | 0.99 (0.97, 1.01) | 0.20 | 1.01 (0.99, 1.02) | 0.34 |
| AMH | 1.01 (1.00, 1.02) | 0.04 | 0.93 (0.88, 1.00) | 0.04 | 1.00 (0.98, 1.02) | 0.83 | 0.92 (0.90, 0.95) | <0.01 |
| Male age | 1.03 (1.01, 1.05) | 0.01 | 1.07 (1.04, 1.10) | <0.01 | 1.01 (1.00, 1.02) | 0.05 | 1.07 (1.06, 1.09) | <0.01 |
| Teratozoospermia | 1.11 (0.75, 1.65) | 0.59 | 1.11 (0.73, 1.69) | 0.63 | 1.18 (0.96, 1.45) | 0.12 | 1.00 (0.79, 1.28) | 0.97 |
| Total Gn dosage ^*^ | 0.71 (0.35, 1.47) | 0.36 | 6.14 (2.27, 16.61) | <0.01 | 1.69 (1.15, 2.48) | <0.01 | 3.84 (2.35, 6.28) | <0.01 |
| No. of oocytes retrieved | 0.99 (0.98, 1.01) | 0.39 | 0.97 (0.96, 0.99) | <0.01 | 0.99 (0.99, 1.00) | 0.16 | 0.98 (0.97, 0.98) | <0.01 |
| Blastocyst day ^a^ | 1.20 (1.00, 1.45) | 0.05 | 1.15 (0.94, 1.39) | 0.17 | 1.07 (0.97, 1.17) | 0.19 | 1.33 (1.20, 1.48) | <0.01 |
| Blastocyst expansion stage ^b^ | 1.31 (0.93, 1.85) | 0.12 | 1.41 (0.96, 2.06) | 0.08 | 1.36 (1.13, 1.63) | <0.01 | 1.25 (1.02, 1.52) | 0.03 |
| ICM grade ^b^ | 1.89 (1.16, 3.09) | 0.01 | 0.80 (0.52, 1.21) | 0.29 | 1.28 (1.04, 1.58) | 0.02 | 1.42 (1.10, 1.84) | <0.01 |
| TE grade ^b^ | 1.11 (0.93, 1.33) | 0.26 | 1.19 (0.99, 1.43) | 0.06 | 1.34 (1.22, 1.47) | <0.01 | 1.47 (1.33, 1.63) | <0.01 |
| Recurrent implantation failure | 1.41 (0.88, 2.26) | 0.16 | 0.88 (0.40, 1.91) | 0.74 | 1.13 (0.99, 1.29) | 0.06 | 0.98 (0.83, 1.16) | 0.83 |
| History of chorionic  chromosomal abnormalities | 0.85 (0.69, 1.05) | 0.14 | 1.15 (0.88, 1.50) | 0.31 | 0.94 (0.84, 1.05) | 0.26 | 1.09 (0.95, 1.24) | 0.21 |

Notes: OR, odds ratio; CI, confidence interval; Gn, gonadotropin; ICM, inner cell mass; TE, trophectoderm.

ᵃ Comparison between Day 6 and Day 5 blastocysts. ᵇBlastocyst expansion compares stages 3+4 vs 5+6; ICM grade compares grade B vs grade A; TE grade compares grade C vs grades A+B. * E2 level and Total Gn dose is expressed in log-transformed values.

Supplementary Table 5 Univariate Generalized Estimating Equation (GEE) Regression Analysis of Factors Associated with Mosaic Embryo Formation in Patients With and Without Recurrent Implantation Failure Undergoing PGT-A

| Variables | Recurrent Implantation Failure Subgroup† | | | | Non-Recurrent Implantation Failure Subgroup | | | |
| --- | --- | --- | --- | --- | --- | --- | --- | --- |
|  | Mosaic vs. Euploid | | Aneuploid vs. Euploid | | Mosaic vs. Euploid | | Aneuploid vs. Euploid | |
|  | OR (95% CI) | *P* value | OR (95% CI) | *P* value | OR (95% CI) | *P* value | OR (95% CI) | *P* value |
| Advanced maternal age | 1.10 (0.74, 1.65) | 0.64 | 1.91 (1.20, 3.06) | <0.01 | 1.14 (0.94, 1.37) | 0.18 | 1.96 (1.60, 2.41) | <0.01 |
| Female BMI | 1.04 (1.00, 1.08) | 0.05 | 0.97 (0.93, 1.02) | 0.29 | 0.99 (0.97, 1.01) | 0.17 | 1.01 (0.99, 1.04) | 0.23 |
| Basal FSH | 1.00 (0.94, 1.06) | 0.96 | 1.07 (0.99, 1.15) | 0.08 | 1.00 (0.99, 1.00) | 0.07 | 0.98 (0.95, 1.02) | 0.42 |
| Basal LH | 0.99 (0.98, 1.01) | 0.37 | 1.00 (0.98, 1.03) | 0.86 | 1.00 (0.99, 1.01) | 0.84 | 1.00 (0.98, 1.02) | 0.86 |
| Basal E_2_^*^ | 0.77 (0.63, 0.94) | 0.01 | 1.02 (0.78, 1.33) | 0.89 | 1.10 (0.98, 1.22) | 0.10 | 0.92 (0.78, 1.08) | 0.32 |
| Basal P | 0.99 (0.96, 1.01) | 0.30 | 1.01 (0.98, 1.03) | 0.67 | 0.99 (0.97, 1.01) | 0.44 | 1.01 (0.99, 1.03) | 0.23 |
| AMH | 1.00 (0.96, 1.03) | 0.80 | 0.91 (0.85, 0.98) | <0.01 | 1.01 (1.00, 1.03) | 0.05 | 0.96 (0.93, 0.98) | <0.01 |
| Male age | 1.00 (0.97, 1.03) | 0.99 | 1.03 (1.00, 1.06) | 0.05 | 1.00 (0.99, 1.01) | 0.91 | 1.06 (1.04, 1.08) | <0.01 |
| Teratozoospermia | 0.91 (0.53, 1.59) | 0.75 | 1.95 (1.07, 3.53) | 0.03 | 1.19 (0.96, 1.47) | 0.11 | 0.92 (0.71, 1.19) | 0.54 |
| Total Gn dosage ^*^ | 1.61 (0.73, 3.56) | 0.24 | 3.13 (1.08, 9.10) | 0.04 | 0.94 (0.62, 1.42) | 0.76 | 2.60 (1.51, 4.48) | <0.01 |
| No. of oocytes retrieved | 1.01 (0.99, 1.02) | 0.32 | 0.96 (0.94, 0.98) | <0.01 | 1.00 (0.99, 1.00) | 0.39 | 0.99 (0.98, 1.00) | 0.02 |
| Blastocyst day ^a^ | 1.11 (0.90, 1.37) | 0.32 | 1.18 (0.92, 1.51) | 0.19 | 1.08 (0.98, 1.19) | 0.13 | 1.31 (1.17, 1.47) | <0.01 |
| Blastocyst expansion stage ^b^ | 1.61 (1.07, 2.42) | 0.02 | 1.74 (0.98, 3.09) | 0.06 | 1.27 (1.06, 1.53) | 0.01 | 1.15 (0.94, 1.41) | 0.18 |
| ICM grade ^b^ | 1.22 (0.77, 1.93) | 0.40 | 2.07 (0.99, 4.33) | 0.05 | 1.39 (1.11, 1.74) | <0.01 | 1.16 (0.90, 1.50) | 0.26 |
| TE grade ^b^ | 1.26 (1.03, 1.55) | 0.02 | 1.44 (1.12, 1.86) | <0.01 | 1.28 (1.17, 1.41) | <0.01 | 1.38 (1.24, 1.53) | <0.01 |
| Recurrent miscarriage | 1.17 (0.74, 1.85) | 0.51 | 0.91 (0.44, 1.87) | 0.80 | 0.94 (0.83, 1.08) | 0.39 | 1.03 (0.87, 1.23) | 0.71 |
| History of chorionic  chromosomal abnormalities | 0.84 (0.55, 1.28) | 0.43 | 1.44 (0.85, 2.46) | 0.18 | 0.93 (0.82, 1.04) | 0.19 | 1.13 (0.98, 1.31) | 0.09 |

Notes: OR, odds ratio; CI, confidence interval; Gn, gonadotropin; ICM, inner cell mass; TE, trophectoderm.

ᵃ Comparison between Day 6 and Day 5 blastocysts. ᵇBlastocyst expansion compares stages 3+4 vs 5+6; ICM grade compares grade B vs grade A; TE grade compares grade C vs grades A+B. * E2 level and Total Gn dose is expressed in log-transformed values.
